# A Newly Defined and Xeno-Free Culture Medium Supports Every-Other-Day Medium Replacement in the Generation and Long-Term Cultivation of Human Pluripotent Stem Cells

**DOI:** 10.1371/journal.pone.0161229

**Published:** 2016-09-08

**Authors:** Behnam Ahmadian Baghbaderani, Xinghui Tian, Jean Scotty Cadet, Kevan Shah, Amy Walde, Huan Tran, Don Paul Kovarcik, Diana Clarke, Thomas Fellner

**Affiliations:** Lonza Walkersville, Inc., Walkersville, MD, United States of America; Universidade de São Paulo, BRAZIL

## Abstract

Human pluripotent stem cells (hPSCs) present an unprecedented opportunity to advance human health by offering an alternative and renewable cell resource for cellular therapeutics and regenerative medicine. The present demand for high quality hPSCs for use in both research and clinical studies underscores the need to develop technologies that will simplify the cultivation process and control variability. Here we describe the development of a robust, defined and xeno-free hPSC medium that supports reliable propagation of hPSCs and generation of human induced pluripotent stem cells (hiPSCs) from multiple somatic cell types; long-term serial subculturing of hPSCs with every-other-day (EOD) medium replacement; and banking fully characterized hPSCs. The hPSCs cultured in this medium for over 40 passages are genetically stable, retain high expression levels of the pluripotency markers TRA-1-60, TRA-1-81, Oct-3/4 and SSEA-4, and readily differentiate into ectoderm, mesoderm and endoderm. Importantly, the medium plays an integral role in establishing a cGMP-compliant process for the manufacturing of hiPSCs that can be used for generation of clinically relevant cell types for cell replacement therapy applications.

## Introduction

Human pluripotent stem cells (hPSCs) include both human embryonic stem cells (hESCs) and hiPSCs. Multiple molecular and functional studies have reported that both hESCs and hiPSCs share the basic characteristics of stem cells, including self-renewal capacity (i.e. the potential to divide indefinitely) and pluripotency (i.e. differentiation into ectoderm, mesoderm and endoderm if given the appropriate environmental cues) [1–3]. Both hESCs and hiPSCs can be utilized as an allogeneic cell source to treat human degenerative disease. Although not guaranteed, hiPSCs hold the potential to produce therapeutic cells for a patient from the patient’s own somatic cells [4].

In order for hPSCs to be used in cell therapy applications, it is critical to establish a robust, reproducible and cGMP-compliant cell culture system. To achieve this goal and avoid lot-to-lot variability, potential regulatory and safety concerns, and scalability challenges, it is recommended to use a defined, feeder-free, and xeno-free reprogramming and cell culture system in the manufacturing process. Recently, the development of defined hPSC growth medium that is not dependent on undefined serum or serum components has substantially reduced variation in some culture system components [5–10].

Despite the recent advances in the development of chemically defined and xeno-free hPSC medium formulations and culture systems [10–16], there are still some challenges that need to be addressed in establishing a robust, reproducible and cGMP-compliant hiPSC manufacturing process. Some of these challenges include: (1) the development of an entirely xeno-free system, starting from somatic cells’ isolation process to hiPSC generation, expansion and final banking; (2) use of non-enzymatic method for serial subculturing of hPSCs in the form of cell aggregates to maintain the integrity of PSC microenvironment [12] and avoid potential chromosomal abnormality; and (3) robustness and reliability of the cell culture system to generate human iPSCs from different tissue sources. Moreover, the processes of generating and maintaining high quality hiPSCs for controlled experimentation or to test and develop clinical therapeutics is labor-intensive. Daily basal media changes and re-application of growth factors and supplements are critical for maintaining pluripotent and genetically stable hPSCs *in vitro*. However, current hPSC media formulations [11,17–19] require or recommend the daily application of medium and supplements to generate high quality hiPSCs or maintain the pluripotency of a proliferating and expanding hPSC population. This not only consumes resources and time but also increases the risk of introducing contaminants. Finally, some of the existing growth media are comprised of a high concentration of growth factors and cytokines [17–19] that may impact the differentiation potential of pluripotent stem cells into specific lineages. Therefore, it is important to use optimal concentrations and combination of cytokines that promote PSC growth and expansion without the need to utilize high concentrations of essential growth factors.

We set out to develop a robust, defined, xeno-free hPSC culture medium that utilizes defined and optimized medium components to ensure a high level of reproducibility in hiPSC generation and hPSC cultivation. Taking a step-by-step approach, a defined, xeno-free medium was developed for generation and expansion of hPSCs using different experimental design strategies (including full factorial design, partial factorial design and response surface. The final formulation of this medium permits the generation of high quality hiPSCs from multiple somatic cell sources and eliminates the need for daily medium replacement and cell handling. The use of this defined, xeno-free hPSC medium with a defined matrix and passaging solution allows use of the same cultivation components and process for pre-clinical research in the clinical manufacturing of human pluripotent stem cells for therapeutic treatments [20].

## Materials and Methods

### hPSC lines

Human embryonic stem cell (hESC) lines H7 (WA07 (MEF Platform), lot No. FTDL-01), H9 (WA09 (MEF Platform), lot No. DL-10) and H14 (WA14 (MEF Platform), lot No. DL-01) were obtained from WiCell (Madison, WI) and initially cultivated on mitomycin-C treated mouse embryonic fibroblasts (MEFs) in 80% Knockout-Dulbecco’s Modified Eagle’s Medium/F12 (Life Technologies, 12660–012), 20% Knockout Serum Replacement (Life Technologies, 10828–028), 2mM L-glutamine (Life Technologies, 25030–081), 55μM β-mercaptoetanol (Life Technologies, 21985–023), 1% non-essential amino acids (Life Technologies, 11140–050), and 8 ng/ml human basic fibroblast growth factor (Life Technologies, PHG0026). NL9, a hiPSC line, was derived on MEFs using a mixture of episomal plasmids containing Oct-3/4, Sox2, Klf4, c-Myc and Lin28. The hiPSC line 18R, used in the long-term evaluation of the medium, was derived on defined L7^™^ hPSC matrix (Lonza, FP-5020) using the same reprogramming plasmids listed above for NL9. LiPSC ER2.2 was generated using a cGMP compliant manufacturing process involving the same reprogramming method and L7 cell culture system as described elsewhere [20]. In the initial medium formulation studies, WA07, WA09 and NL9 were adapted to feeder-independent conditions using hESC-qualified Matrigel^**™**^ (Becton Dickinson). In the long-term evaluation of the defined, xeno-free EOD L7™ hPSC medium, WA07, WA09, WA14 and NL9 were adapted and continuously maintained in feeder-independent conditions on defined L7^**™**^ hPSC Matrix (Lonza, FP-5020). L7™ hPSC Matrix is a proprietary, chemically defined recombinant protein and commercially available product. L7™ hPSC Passaging Solution (Lonza, FP-5013), a commercially available, non-enzymatic passaging solution based on sodium citrate, was used for serial subculture of hPSCs in the form of cell aggregates to maintain the integrity of PSC microenvironment [12]. All hPSC lines were cultivated in a humidified 37°C cell incubator equilibrated with 5% CO_2,_ unless noted otherwise_._

### Experimental Design for the Development of Basal Medium and supplement formulation

A step-by-step approach was taken to develop a basal medium and growth supplement. Briefly, we first evaluated different formulations of basal medium specifically designed to support the expansion of human pluripotent stem cells. Then, we focused on the development and optimization of a medium supplement. The medium components and cytokines were evaluated using design of experiment (DOE) methods, including partial factorial design (six components with 32 conditions plus center point), response surface (three variables with 14 conditions plus the center point), and full factorial design (two factors with four levels). Each culture condition was qualitatively and quantitatively evaluated based on a score system reflecting the cell attachment, cell morphology, cell growth and spontaneous differentiation.

### Determination of bFGF stability in the defined medium formulation

The stability of native bFGF and thermostable (TS) bFGF were compared to determine their stability in the defined L7^**™**^ hPSC medium formulation. WA09 cells were seeded at 2 x 10^5^ cells per well in a 6-well tissue culture treated plate coated with L7^**™**^ hPSC matrix. The defined hPSC medium was replaced daily for two days. Three days post-seeding, the medium of metabolically active hPSC cultures was replaced with freshly supplemented basal medium containing either bFGF or TS bFGF, with or without porcine heparin. Twenty four and 48 hours post-addition, a sample of the medium was taken and the concentration of bFGF present determined using the Quantikine ELISA for hbFGF (RND Systems, DFB50).

### Generation of hiPSCs using the defined, xeno-free EOD hPSC medium

Cryopreserved human umbilical Cord Blood (hUCB) CD34^+^ cells (Lonza, 2C-101) were thawed and expanded in StemSpan^**™**^-ACF (STEMCELL™ Technologies, 09805) supplemented with 100 ng/mL recombinant human (rh)SCF (PeproTech, AF-300-07), 100 ng/ml rhFlt3-ligand (PeproTech, AF-300-19), 20 ng/ml rhThrombopoietin (PeproTech, 300–18) and 10 ng/ml IL-3 (PeproTech, 200–03). The CD34^+^ cells were seeded in 12-well plates (Corning, 3513) at a density of 4–6 ×10^5^ cells per well. Confluent cells (approximately day 3 post-thaw) were passaged the day prior to Nucleofection. Cryopreserved human Peripheral Blood Mononuclear Cells (hPBMCs) (Lonza, CC-2702) were expanded as described previously [21]. Cryopreserved human adult dermal fibroblasts (hDFs) (Lonza, CC-2511) were thawed and maintained in chemically defined dermal fibroblast cell growth medium (Lonza, 00199041) following the manufacturer’s instructions.

1×10^6^ hUCB CD34^+^ cells or 2×10^6^ hPBMCs were reprogramed using the episomal plasmids encoding Oct4, Sox2, Klf4, c-Myc and Lin28 and pEB-Tg [21]. These plasmids were introduced into the cells using the 4D-Nucleofector^**™**^ System and P3 solution Kit (Lonza, V4XP-3012). After nucleofection, cells were plated back in their respective expansion medium in a 37°C humidified incubator containing 5% CO_2_ and 3–5% O_2_. Thirty micrograms of aluminium hydroxide gel (Alhydrogel^®^, InvivoGen, vac-alu-250) were immediately supplemented into the expansion medium to enhance the reprogramming efficiency if necessary. Two days later, the cells were transferred onto L7^**™**^ hPSC Matrix-coated 6-well plates in the defined, xeno-free EOD hPSC medium supplemented with 1μM TGFβ inhibitor (Stemgent, 04–0014). Cells were placed in a 37°C humidified incubator containing 5% CO_2_ and 3–5% O_2_. Medium was changed EOD.

Human dermal fibroblasts reprogramming was carried out using a combination of 5 vectors: pCE-hOCT3/4 (Addgene, 41813), pCE-hSK (Addgene, 41814), pCE-hUL (Addgene, 41855), pCE-mp53DD (Addgene, 41856) and pCXB-EBNA1 (Addgene, 41857) [22,23]. Briefly, these plasmids were introduced into hDFs using the 4-D nucleofection system and P3 solution kit. After nucleofection, hDF cells were maintained in DMEM (Life Technologies, 11965–092) medium supplemented with 10% FBS for 7 days. The cells were then dissociated and re-plated in L7^**™**^ hPSC EOD Medium on L7^**™**^ hPSC matrix-coated plates. Cells were cultured in in a 37°C humidified incubator containing 5% CO_2_ and 3–5% O_2_ with EOD medium changes.

### Human donor cells

All cells utilized in this study were isolated from donated human tissue after obtaining permission for their use in research applications by informed consent or legal authorization.

### Immunocytochemistry of hPSCs maintained in the defined, xeno-free L7^**™**^ hPSC EOD Medium

Human PSCs were cultured in the culture medium indicated. Colonies present in the cultures on days 3 through 5 were prepared for immunocytochemical analysis. The culture medium was aspirated and washed twice with 1X Dulbecco’s Phosphate Buffered Saline (Lonza Biosciences, 17-513F). The cells were fixed in 1X DPBS containing 4% PFA (Electron Microscopy Sciences, 15710) for 20 minutes, then rinsed twice with PBS-T (0.2% Tween-20 in 1X DPBS) for 5 minutes (Sigma-Aldrich, P9416), followed by a 2 hour incubation with 10% donkey serum in PBS-T at room temperature. The hPSCs were then treated with primary antibodies detecting extracellular antigens SSEA4 (Millipore, MAB4304; 1:100), TRA-1-60 (Millipore, MAB4360; 1:100) and TRA-1-81 (StemGent, 09–0011; 1:100) overnight at 2–8°C prior to being permeabilized for 20 minutes in 1X DPBS containing 0.1% Triton X-100 (Sigma-Aldrich, T9284). A second blocking step with 10% donkey serum solution was performed before incubating the cells with intracellular primary antibodies overnight at 2–8°C. Primary antibodies raised against pluripotency-associated antigens OCT4 (Abcam, ab19857; 1:350) and Nanog (R&D Systems, AF1997; 6.7μg/ml) were used in combination with the secondary antibodies Cy3-conjugated Donkey anti-rabbit IgG (Jackson ImmunoResearch, 711-165-152; 1:200) and Cy3-conjugated donkey anti-Goat IgG (H+L) (Jackson ImmunoResearch, 805-165-180; 1:200), respectively. Primary antibodies specific for SSEA4 and TRA-1-60/TRA-1-81 were used in combination with secondary antibodies Alexa Fluor 488-cojugated donkey anti-mouse IgG (H+L) (Jackson Immunoresearch, 715-545-150; 1:200) and Alexa Fluor 488-cojugated donkey anti-mouse IgM (H+L) (Jackson Immunoresearch, 715-545-140; 1:200), respectively. All cells were incubated with secondary antibodies for 2 hours and then counterstained with 300 nM DAPI (Life Technologies, D3571) in 1X DPBS at room temperature for 15–30 minutes. Cells were rinsed after permeabilization and between the incubation of the primary and secondary antibodies. 50% Glycerol was immediately added to the wells after the final wash with PBS-T. All fluorescence detection was visualized using an EVOS FL all-in-one microscope equipped with software version 17625.

### Embryoid body differentiation

Confluent cultures of WA09 hESC colonies were dissociated using L7^**™**^ hPSC Dissociation Solution. Cell aggregates were suspended in EB formation medium consisting of DMEM/F12 (Life Technologies, 11330–032) containing 10μM Rock Inhibitor Y27632 (Millipore, SCM075) and allowed to settle by gravity in a conical tube. After removing the supernatant, cells were suspended in fresh EB medium. Cell aggregates were then seeded using a split ratio of 1:1 on Ultra Low Attachment (Corning, 3471) plates and returned to the incubator for 12 to 24 hours. Once large cell aggregates formed, they were collected into a conical tube and allowed to settle by gravity. The medium was then removed and replaced with differentiation medium (80% DMEM High Glucose (Life Technologies, 11965–092), 20% defined fetal bovine serum (Hyclone, SH30070.03), 1X non-essential amino acids (Life Technologies, 11140–050), 2 mM L-glutamine (Cellgro/Mediatech, 25-005-CI) and 55 μM β-Mercaptoethanol (Life Technologies, 21985–023)). Cell aggregates were placed on Ultra Low Attachment plates using a split ratio of 1:1 in 0.4 ml differentiation medium/cm^2^. Culture medium was changed every second day for six days. On the seventh day, EBs were seeded on gelatin-coated plates (EmbryoMax^®^ ES Cell Qualified Gelatin Solution (Millipore, ES006-B)) at approximately 10 EBs/cm^2^. The EBs were allowed to attach undisturbed for 2 days. The differentiation medium was changed after the second day and every other day afterward with 0.4 ml/cm^2^ differentiation medium. The cultures were prepared for immunocytochemistry on day 14–15.

The differentiated hPSCs were fixed with 4% PFA and permeabilized with 0.1% Triton X-100 PBS solution as described above. After rinsing the fixed cells with PBS-T solution, the cells were incubated with DPBS containing 10% goat serum (Life Technologies, 10000C) for 2 hours at room temperature. Primary antibodies detecting alpha-1 Fetoprotein (Abcam, ab3980; 1:200 or R&D systems, MAB1369, 1:100), beta III tubulin (Millipore, MAB1637; 1:400) and Smooth Muscle Actin (DAKO, M0851; 1:500) were incubated overnight at 2–8°C. Cells were rinsed twice with PBS-T, and the secondary antibody, Alexa Fluor 488-conjugated goat anti-mouse IgG(H+L) (Life Technologies, A11001; 1:1000) or Alexa Fluor 494-conjugated goat anti-mouse IgG(H+L) (Life Technologies, A-11032; 1:1000) were added and incubated on the cells for at least 2 hours at room temperature. Cultures were then rinsed three times (5 minutes each) in 1X DPBS prior to counterstaining with DAPI. Cells were maintained in 50% glycerol for analysis.

### Flow cytometry

hPSCs were cultured in their respective media until they were approximately 70 to 80% confluent. Cultures were then dissociated into a single-cell suspension using a solution of 0.05% Trypsin/EDTA (CellGro, 25-052-CI) containing 2% chick serum (Sigma-Aldrich, C5405). Cells were fixed and permeabilized for intracellular staining with the Cytofix/Cytoperm Kit (Becton Dickinson, 554714) following the manufacturer’s recommended protocol. Permeabilized cells were incubated with PE-conjugated anti-OCT3/4 (R&D Systems, IC1759P) or respective PE-conjugated rat IgG2b isotype control.

Extracellular antigens were detected on unfixed cells stained with PE-conjugated antigen-specific antibodies and respective isotypes using the manufacturer’s recommended concentration: anti-TRA-1-60 (BD Biosciences, 560193), anti-TRA-1-81 (BD Biosciences, 560161), mouse IgG3 isotype (BD Biosciences, 556659); anti-SSEA4 (BD Biosciences, 560128) and mouse IgM isotype (BD Biosciences, 555584). Samples were processed through a FACS Calibur (Becton Dickinson) or FACSCanto^**™**^ II flow cytometer. Data were acquired using CellQuest Pro 5.2.1 or BD FACS Diva software and analyzed with Flowjo 7.6 software.

### Karyotype, CGH and SNP analysis

To determine if any genetic abnormalities were present after the hPSCs were generated or continuously passaged in the defined, xeno-free L7^™^ hPSC EOD medium, exponentially proliferating cultures from each independent culture were prepared on or after passage number 40 and sent to Cell Line Genetics (Madison, Wisconsin). Cytogenetic analysis was performed on a minimum of 20 G-banded metaphase cells for each independent sample. In addition, hiPSC lines generated in the defined, xeno-free L7^™^ hPSC EOD medium were evaluated using a comparative genomic hybridization (CGH) and Single Nucleotide Polymorphism (SNP) Microarray. CGH and SNP analysis (WiCell Cytogenetics, Madison Wisconsin) was performed on each line to confirm the lines were genetically stable and balanced.

### Comparison between xeno-free L7^™^ hPSC EOD with mTeSR1 and E8 medium

Human pluripotent stem cells (one hiPSC line–LiPSC ER2.2; one hESC line–WA07 line) were plated and passaged using the methods recommended by each manufacturer. Briefly, hPSCs cultured in the defined, xeno-free L7^™^ hPSC EOD medium were incubated with L7^**™**^ hPSC Passaging Solution (Lonza, FP-5013) for 5 to 7 min at room temperature and plated on L7^**™**^ hPSC Matrix-coated (Lonza, FP-5020) plates. Culture medium was initially changed on day 1 after seeding but thereafter, only EOD. The cells cultured in Essential 8^**™**^ Medium (Life Technologies, A1517001) were incubated with 0.5mM EDTA (Life Technologies, 15575–020) for 5–7 min at room temperature and then plated on recombinant human Vitronectin-coated (5 μg/ml) (Life Technologies, A14700) plates. Human PSCs cultured in mTeSR1 Medium (StemCell Technologies, 05850) were serially subcultured using ReLeSR^**™**^ (StemCell Technologies, 05872) on Matrigel^**™**^. The medium was changed daily. Prior to the start of the study, the quality of the cells was evaluated using karyotype analysis and flow cytometry, employing the methods described previously. The cells were seeded in each cell culture system (three replicates per condition) at 2.0 × 10^4^ viable cells seeded per cm^2^, and serially subcultured for at least 5 passages, at which point the cells underwent additional characterization using flow cytometry, EB formation and immunocytochemistry. At the end of each passage, the cells from each of three replicate wells in a 6-well plate were suspended in 3 ml of medium. The viability and total viable cells were calculated using NucleoCounter^®^ NC-200^**™**^ (Chemometec AS).

### Teratoma formation

hPSC colonies, cultured in the defined, xeno-free L7^™^ hPSC EOD medium for 40 cell passages or more, were dissociated using L7^**™**^ hPSC Passaging Solution. Cells were counted, distributed into tubes, and pelleted. Cells were then suspended in knockout DMEM/F12 media containing 5% defined FBS. ≥ 5x10^6^ cells were injected intramuscularly into the right hind leg flank of six week old SCID/Beige mice. Three to five mice were injected per condition. The mice were observed daily, and the tumor measured twice a week and recorded. The tumors were allowed to grow to 1.5 cm in diameter. The mouse was then euthanized, the tumor excised, embedded in buffered formalin and sent to Charles River (Wilmington, Massachusetts) for sectioning and hematoxylin and eosin staining. Slides with representative cell types derived from three germ layers were examined by a certified pathologist. Pathology was also performed on control tissues derived from the non-injected legs of these same mice.

### Animal ethics statement

The SCID/Beige mice (Harlan Laboratories) used to produce the experimental teratomas in this study were housed in Lonza Walkersville, Inc.'s AAALAC accredited (Association for the Assessment and Accreditation of Laboratory Animal Care) animal facility and were cared for in accordance with the principles outlined in the *ILAR Guide for the Care and Use of Laboratory Animals*. The animal use was approved by the (Lonza Walkersville) Institutional Animal Care and Use Committee in accordance with the USDA Animal Welfare Act.

The method of euthanasia was C02 overdose followed by cervical dislocation. The Lonza Animal Program had a written and approved moribund sacrifice policy in effect during the study. None of the experimental animals died prior to reaching the study endpoint.

## Results

**Fig 1:**
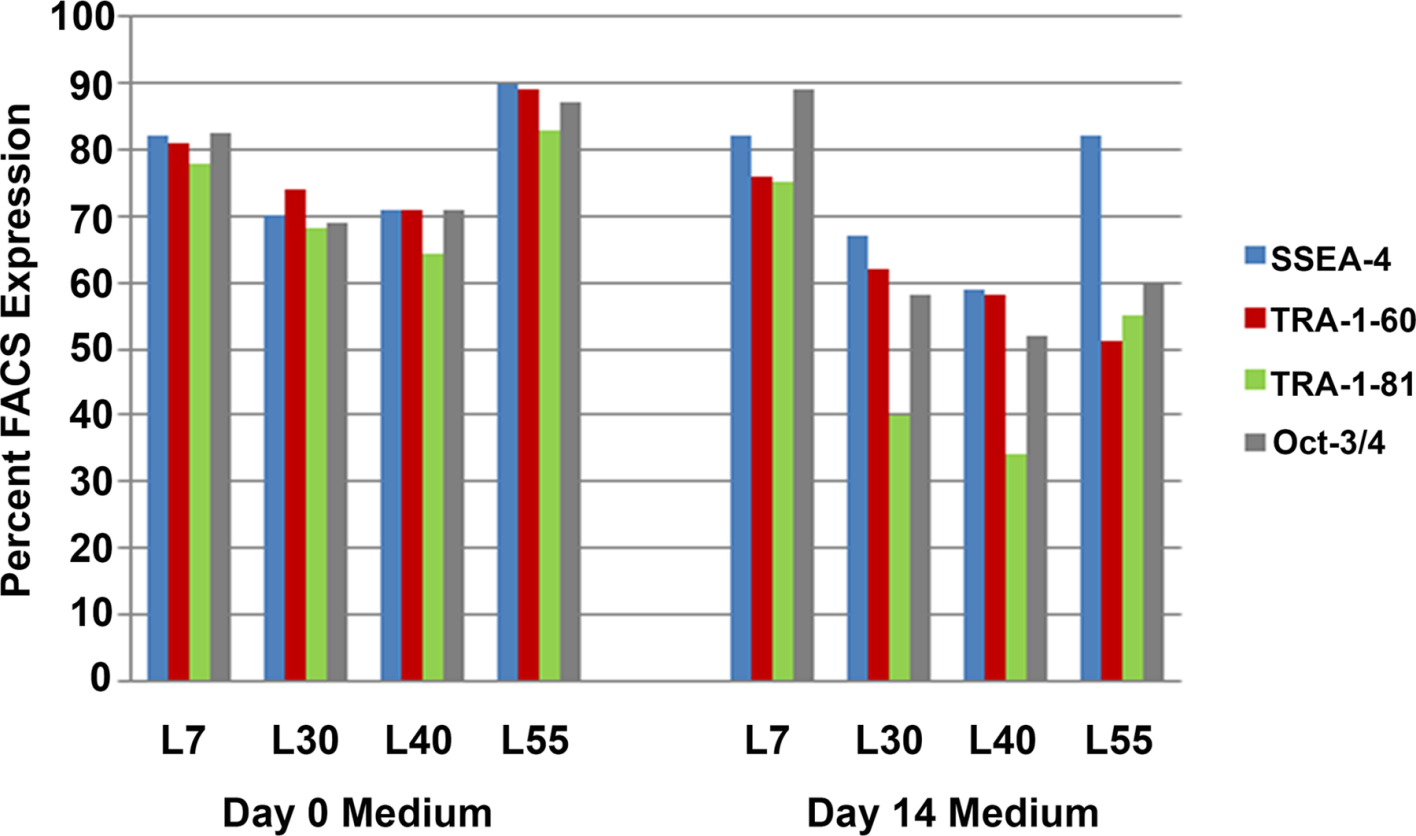
Differences observed in the stability of distinct hPSC medium formulations. Four hPSC medium formulations were found to support the maintenance of hPSC lines in culture. To evaluate the stability of each formulation, both freshly supplemented medium and medium that was supplemented and then stored at 4°C for 14 days prior to addition to the hPSC lines, were compared. In this representative example using WA09 cells, all formulations sustained high expression levels of pluripotency markers when the medium was used within seven days after supplementation with bFGF and other factors. However, only the medium containing the L7 supplement formulation was able to sustain robust expression of the hPSC markers with aged medium (14–21 days) post supplementation.

To develop a defined, xeno-free hPSC medium, basal medium components and supplements were systematically evaluated using a step-by-step approach and different EOD experiments explained in the Materials and Methods section to identify medium formulations that supported hPSC survival, proliferation and self-renewal during routine cultivation. Initially, components of the basal medium and key supplements were evaluated independently on three hESC lines: WA07, WA09 and WA14. These hESC lines were independently cultivated for ten passages and the medium evaluated for its ability to support cell attachment to hESC-qualified matrigel, maintain normal hPSC cell morphology, promote cell growth and deter spontaneous differentiation. Four formulations were found to be superior based on these selection criteria (data not shown). To evaluate the combined stability of their components, each formulation was then aged for two weeks at 4°C and then directly compared to its freshly prepared counterpart (Fig 1). While all four media formulations were initially similar in their ability to promote cell growth, prevent spontaneous differentiation and express markers of pluripotency, only L7 formulation was found to maintain these criteria two weeks post-supplementation.

**Fig 2:**
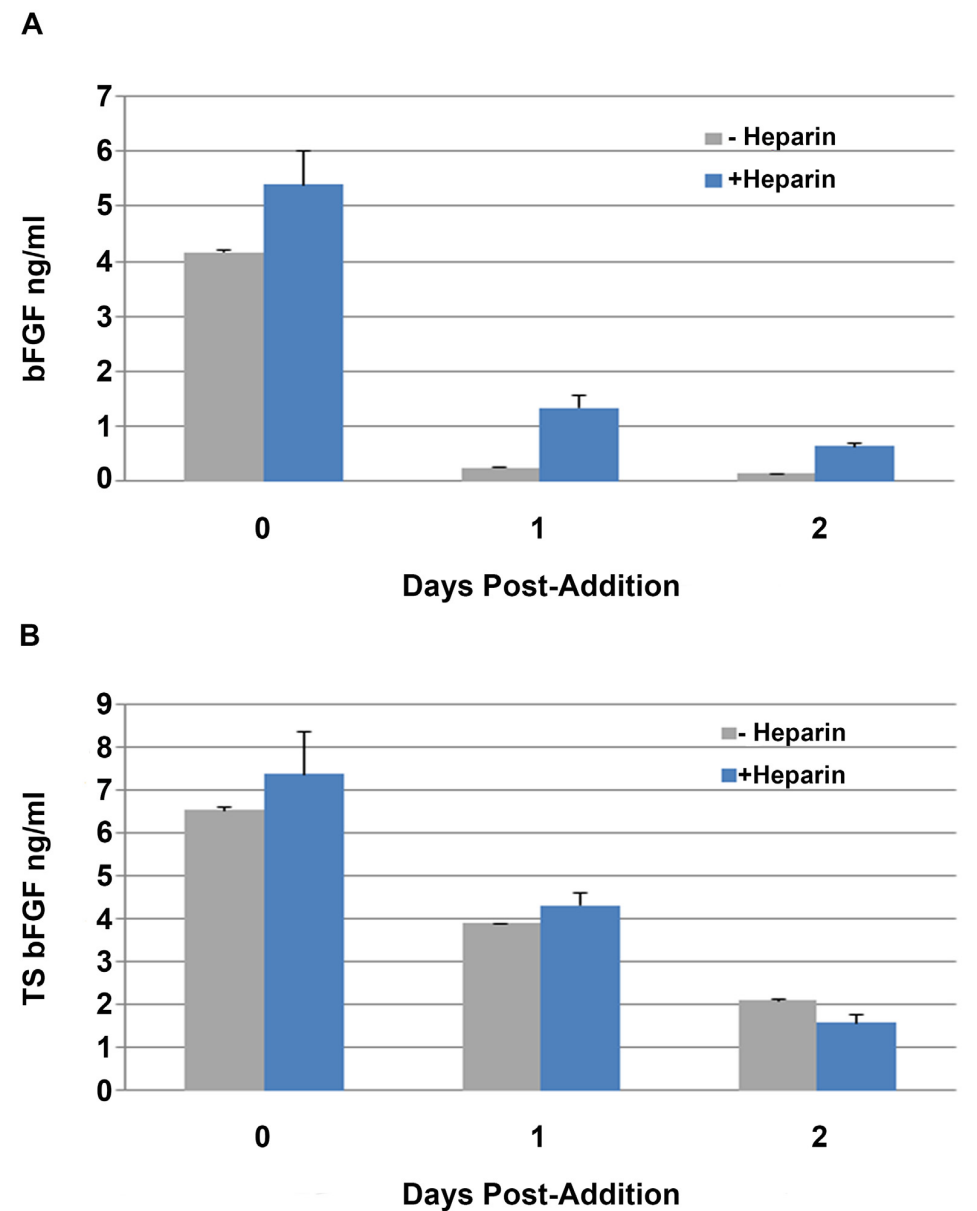
Medium containing thermostable bFGF supports every-other-day supplementation of hPSC cultures. hPSCs (WA09 hESC line) were seeded at 2 x 10^5^ cells per well in a 6-well tissue culture treated coated with L7^**™**^ hPSC matrix in the defined hPSC medium. Three days post-seeding, the medium of metabolically active hPSC cultures was replaced with freshly supplemented basal medium containing either bFGF or TS bFGF. Twenty four and 48 hours post-addition, a sample of the medium was taken and the concentration of bFGF present determined by ELISA. A) bFGF concentrations present in the hPSC medium at the time of addition, 24 hours- and 48 hours-post addition (n = 3). B) TS bFGF concentrations present in the hPSC medium at the time of addition, 24 hours- and 48 hours-post addition (n = 3).

To further optimize this hPSC medium formulation (i.e. L7 formulation), we examined the stability of bFGF present in mitotically active cultures of hPSCs, one and two days post-addition in comparison with a thermo-stable version of bFGF (TS bFGF) (Fig 2A). In both the absence and presence of stabilizing concentrations of porcine-derived heparin, native bFGF concentrations significantly declined 24 hours post-addition and continued to fall to a very minimal level after 48 hours (about %95 decay). In contrast, the level of TS bFGF declined to about 60% and 32% of the initial concentration after 24 and 48 hrs, respectively (Fig 2B). The starting concentration of TS bFGF in this experiment was different from bFGF concentration on day 0, however the concentration was chosen to support a minimum bFGF requirement identified in growth cytokine evaluation studies (data not shown). The quantity of TS bFGF present after 48 hours was comparable to the levels of native bFGF observed 24 hours post-supplementation with daily medium changes. Importantly, TS bFGF was stable in the hPSC medium in the absence of heparin, and therefore heparin could be eliminated from the medium, resulting in the final defined, xeno-free hPSC medium formulation (Table 1).

**Table 1:** The presence and absence of common hPSC medium factors and supplements in the defined, xeno-free EOD hPSC medium.

EOD Basal Medium Supplements	
bFGF	Thermostable bFGF
Activin A	+
Heparin	-
TGFβ1	+
LR-IGF	+
HSA	+
Phenol Red	-

### hPSC expansion in the defined, xeno-free hPSC medium

**Fig 3:**
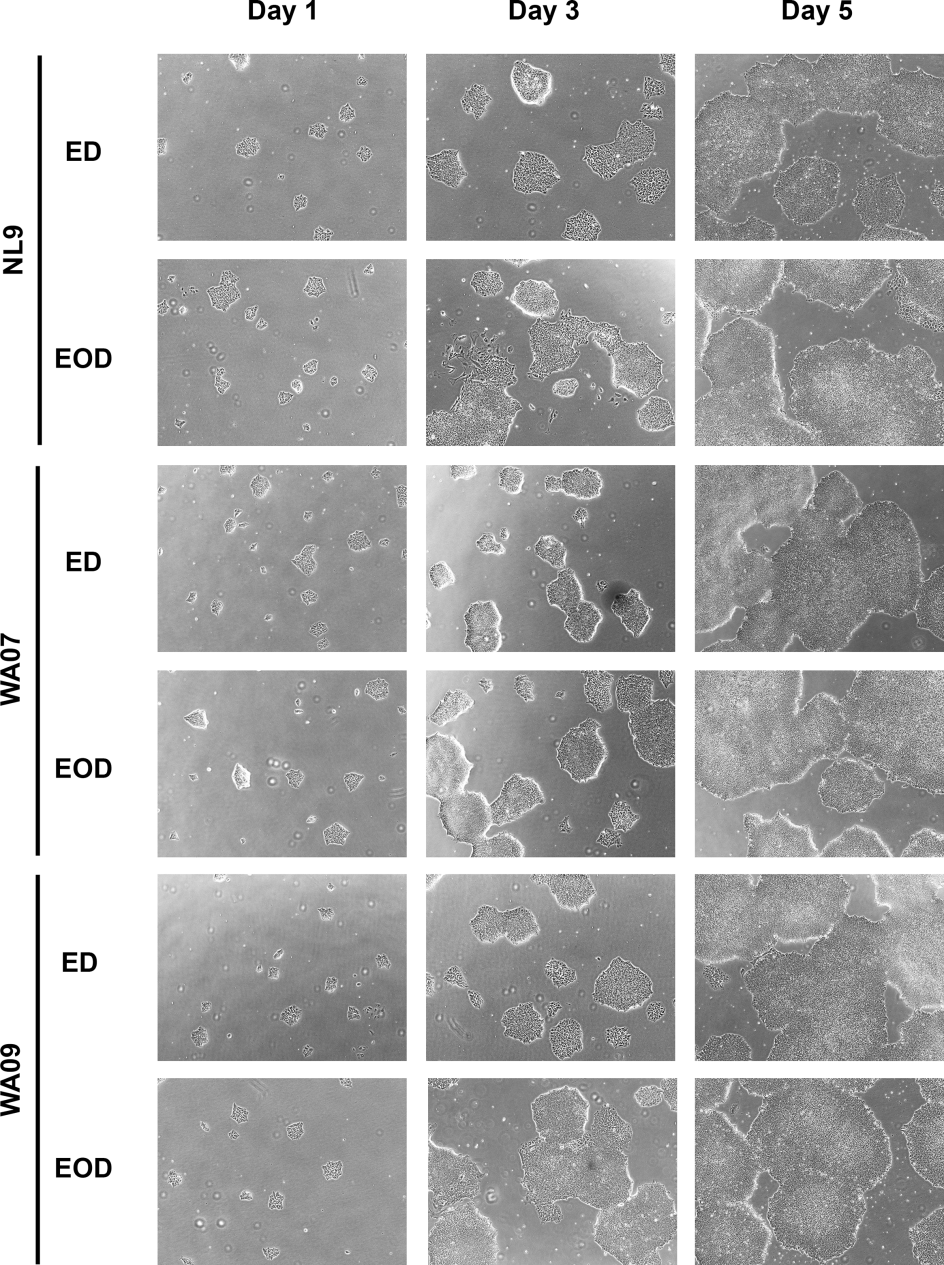
Comparison of hPSC morphology and growth using the defined, xeno-free hPSC medium. Three hPSC lines were adapted to the defined, xeno-free medium formulation supplemented with either bFGF and heparin or TS bFGF without heparin. Cultures seeded at 2 x 10^4^ cells/cm^2^, received either daily medium changes with medium containing heparin and bFGF or received medium containing TS bFGF but no heparin every other day (EOD). Scale bar: 200 μm.

Finally, to test the ability of TS bFGF to sustain the cultivation of hPSCs in the xeno-free medium formulation, we adapted cells to this new medium and tested whether daily supplementation with fresh hPSC medium containing heparin and bFGF performed better than hPSCs supplemented every other day with hPSC medium containing TS bFGF but no heparin (Fig 3). Using a cell seeding ratio of 2 x 10^4^ cells/cm^2^, hPSCs cultivated with daily changes of their culture medium reach confluence on day 5 post-seeding. The hPSC morphology and rate of proliferation observed using TS bFGF were consistent with conditions using native bFGF where the culture medium was changed every day. Only minor changes in the compactness of the cells near the periphery of the colony were observed in the hESCs cultivated in the TS bFGF formulation with EOD medium changes. Importantly, no significant changes were observed in the expression of markers of pluripotency, the amount of spontaneous differentiation or in the cells ability to differentiate into cells representing early embryonic ectoderm, mesoderm and endoderm. It should be noted that when bFGF was used in EOD feeding strategy in the presence or absence of heparin, the bFGF level was not sufficient to support PSC growth and induced significant spontaneous differentiation (data not shown).

### Long term stability of hPSCs cultivated in the defined, xeno-free EOD hPSC medium

**Fig 4:**
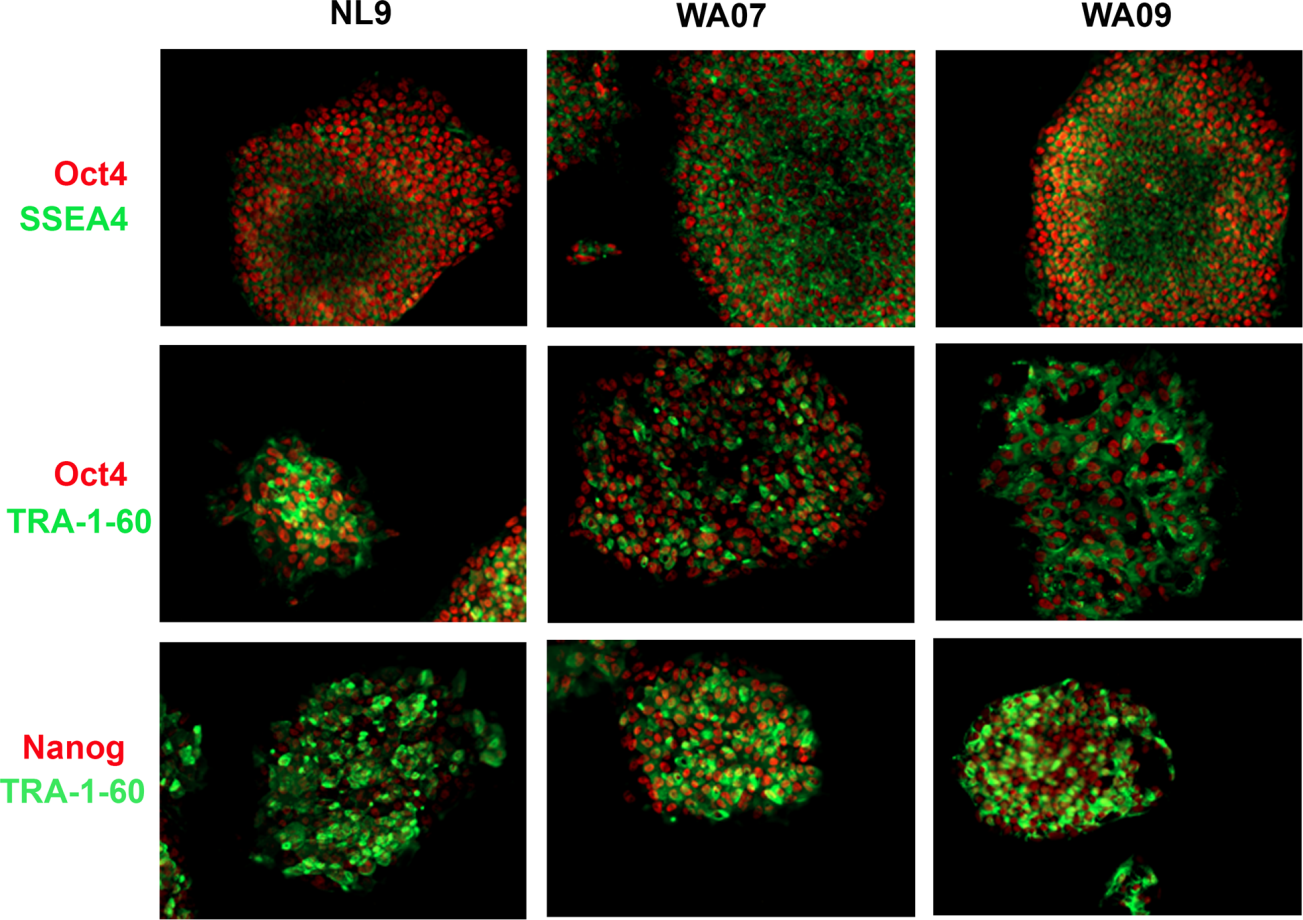
hPSCs cultured in the defined, xeno-free EOD hPSC medium retain their stem cell characteristics. Immunodetection of pluripotency antigens in hPSC lines cultivated for 40 passages in the defined, xeno-free L7 hPSC medium with EOD medium changes. Detection of Oct-3/4 and Nanog are shown (red) along with SSEA-4 and TRA-1-60 (green). Individual cell nuclei were visualized using DAPI (blue). Scale bar: 200 μm.

**Fig 5:**
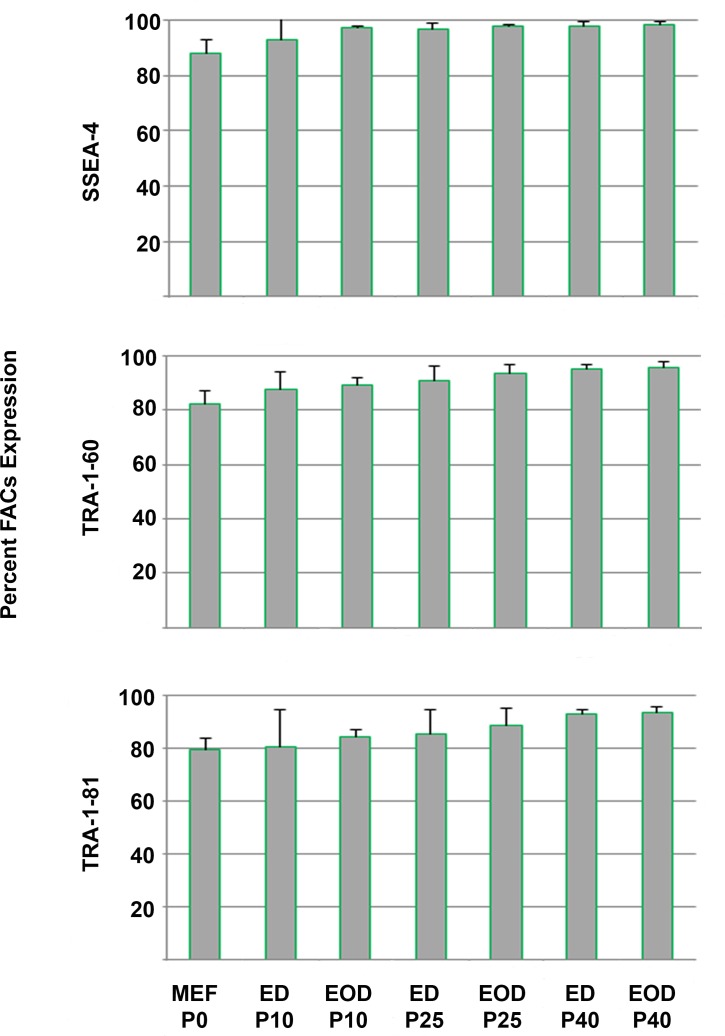
Expression of pluripotency markers in hPSCs cultured for 40 passages using the defined, xeno-free EOD hPSC medium. Three hPSC lines (two replicates per line) were evaluated for their ability to sustain the expression of markers of pluripotency at passage 10, passage 25 and passage 40. Each hPSC line was independently analyzed by flow cytometry for the expression of SSEA-4, TRA-1-60 and TRA-1-81. Results within each passage were combined and the mean value of marker expression determined. Bars represent mean percentage values. Error bars represent the standard deviation of the mean for each passage.

To evaluate the long-term stability of hPSCs cultivated in the defined, xeno-free EOD hPSC medium containing TS-bFGF, two hESC lines (WA07 and WA09) and two hiPSC lines (NL9 and 18R) were continuously cultivated in this medium for over 40 passages. hPSCs maintained in this medium formulation with EOD medium changes for over 40 passages continued to display hESC-like morphology and express high levels of pluripotent stem cell markers (Fig 4). Combined FACS analysis on the hPSC lines showed that a high percentage of the cells expressed antigens for SSEA-4, TRA-1-60 and TRA-1-81 at passages 10, 25 and 40 (Fig 5). The expression of these markers was comparable to those expressed by the cells passaged using medium containing heparin and bFGF, but with every day (ED) medium changes. Importantly, long-term cultivation of the cells in the defined, xeno-free EOD hPSC medium with EOD medium changes had no effect on the maintenance of a normal karyotype, as measured by G-banding analysis (Table 2).

**Table 2:** Characterization of hPSCs generated^*^ and cultured in defined, xeno-free EOD hPSC medium. Percentage of cells expressing markers of pluripotency after cultivation or generation in the defined hPSC medium using flow cytometry; detection of ectoderm, mesoderm and endoderm markers after EB differentiation; results of G-banding, CGH/SNP analyses.

hPSC Line	ID	Tissue Source	SSEA4	Oct3/4	Tra-1-60	Tra-1-81	Tuj1	SMA	AFP	G-Band	CGH/SNP
WA07	hESC	ICM	>90%	>90%	>90%	>90%	+	+	+	normal	N/A
WA09	hESC	ICM					+	+	+	normal	N/A
WA14	hESC	ICM					+	+	+	normal	N/A
NL9	hiPSC	CB CD34^+^					+	+	+	normal	N/A
18R	hiPSC	CB CD34^+^					+	+	+	normal	N/A
34B*	hiPSC	PBMC					+	+	+	normal	normal
44I*	hiPSC	CB CD34^+^					+	+	+	normal	normal
44P*	hiPSC	CB CD34^+^					+	+	+	normal	normal
51H*	hiPSC	hDF					+	+	+	normal	normal

**Fig 6:**
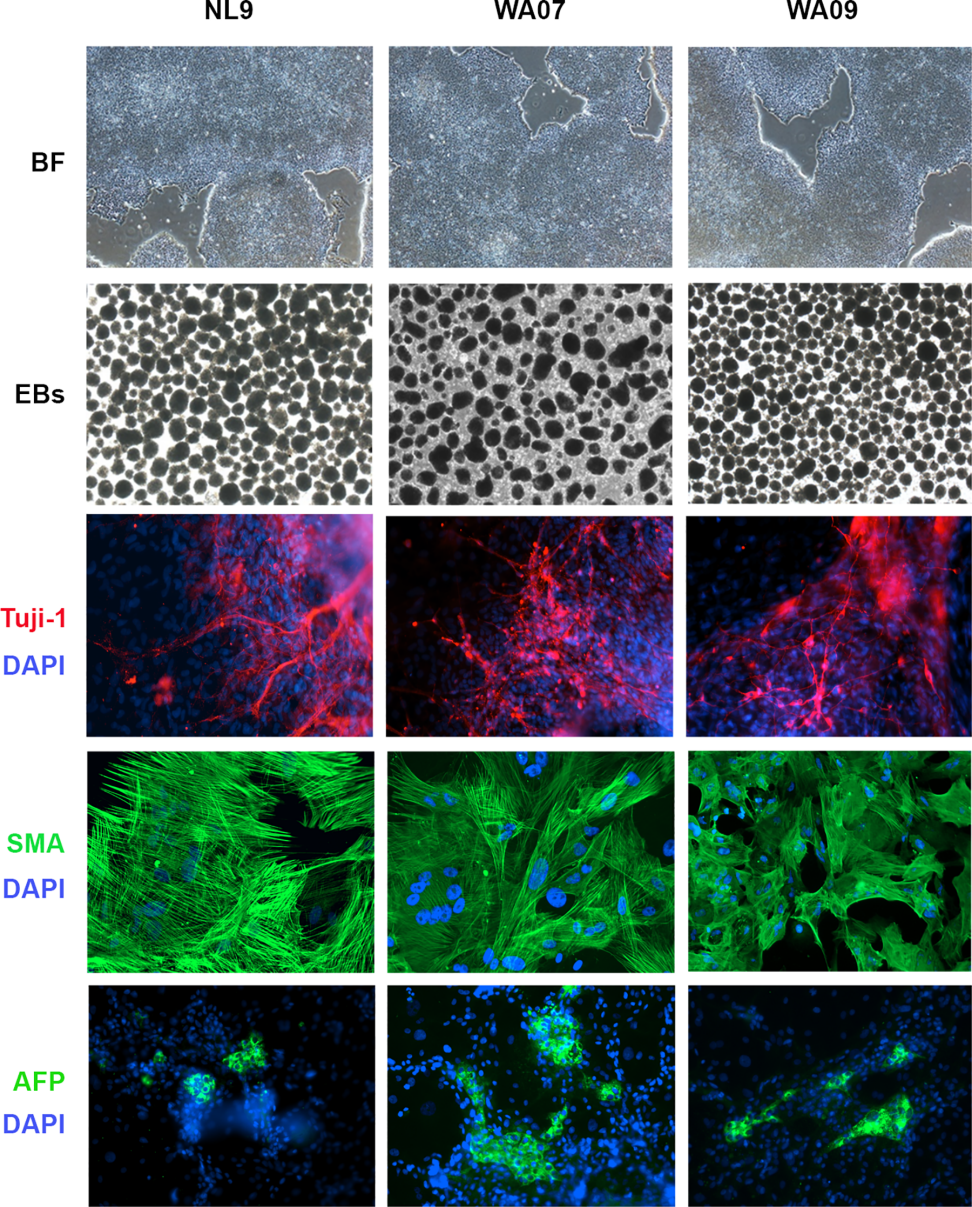
hPSCs cultured for over 40 passages using the defined, xeno-free hPSC medium differentiate readily to early ectoderm, mesoderm and endoderm (EB formation). A) Phase contrast images of the hPSC lines just prior to differentiation. B) Embryoid body formation in cultures differentiated for 14 days in culture. C) Antibodies detecting Beta-III-Tubulin (TUJ1) are shown in red; Smooth Muscle Actin (SMA) and Alpha-Feto Protein (AFP) antigens are shown in green. Nuclei were visualized using DAPI (blue). Scale bar: 200 μm.

To further evaluate the pluripotency of the cell lines cultivated in this defined medium, we assessed their ability to differentiate *in vitro*. After 40 passages, the hPSC lines readily formed embryoid bodies (EBs; Fig 6). Immunocytochemical analysis confirmed the presence of cells expressing class III beta tubulin (Tuj1), smooth muscle actin (SMA), and alpha-feto protein (AFP) representing markers of early embryonic ectoderm, mesoderm and endoderm, respectively. Moreover, teratoma formation analysis demonstrated that both the hESC and hiPSC lines displayed classical histological features of ectoderm, mesoderm and endoderm (S1 Fig).

### hPSC Media comparison study

**Fig 7:**
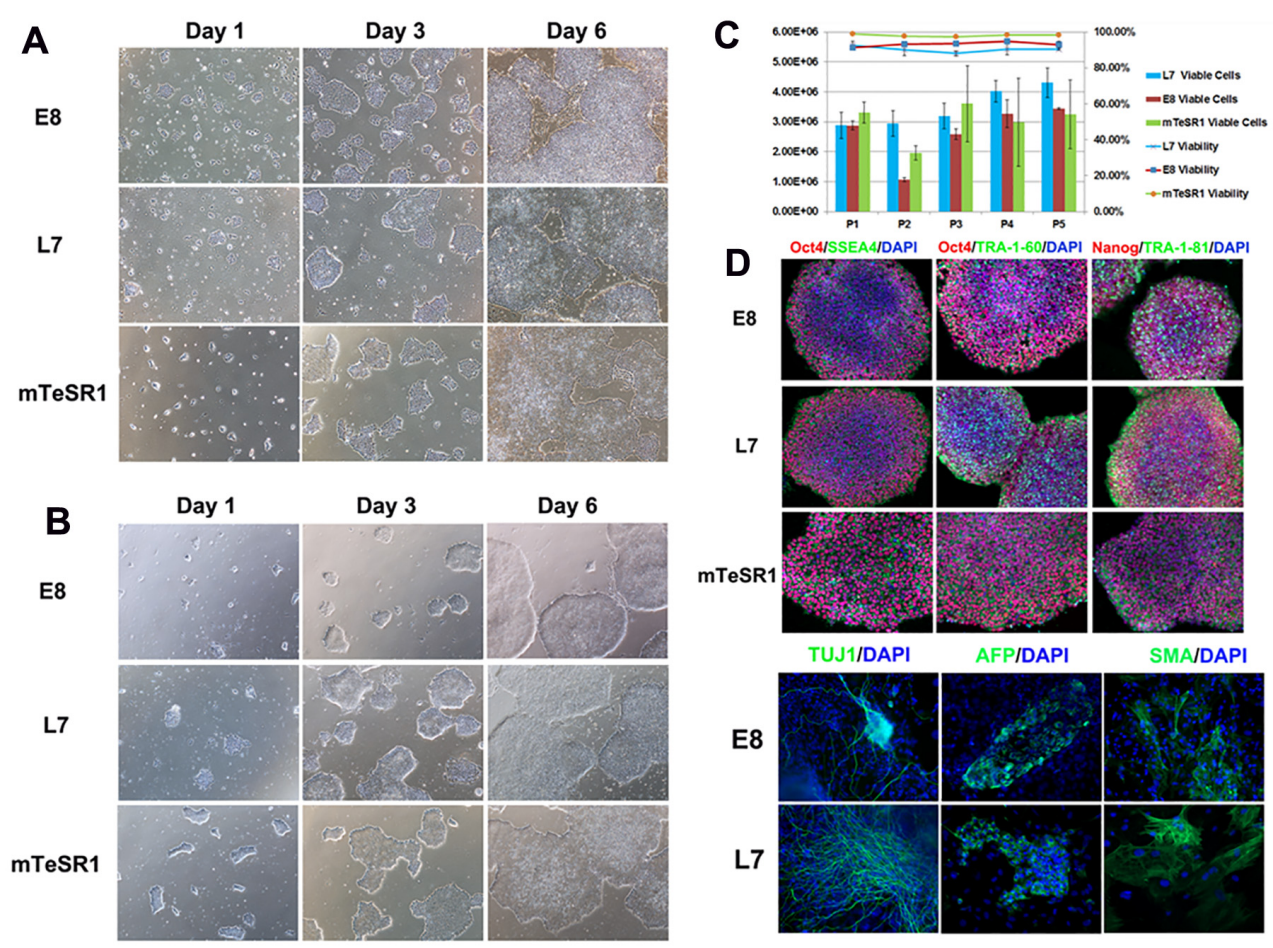
Comparison of the defined, xeno-free EOD L7^™^ hPSC EOD medium to mTeSR1^™^ E8^™^ medium. Human hESC line WA07 was serially subcultured for five passages. The number of viable cells per passage were compared by initially seeding 2×10^4^ viable cells per cm^2^ into three wells of a six-well plate for each medium, using respective passaging solution and matrix. Panel A shows cell attachment and growth of WA07 hESCs at passage 1 in L7^**™**^ hPSC, mTeSR1^TM^, and E8^TM^ media. Panel B shows cell attachment and growth of WA07 hESCs at the end of passage 5 in L7^**™**^ hPSC, mTeSR1^**™**^, and E8^**™**^ media. Panel C Panel A shows the viability and total viable cells of WA07 hESCs grown in L7^**™**^ hPSC, mTeSR1^**™**^, and E8^**™**^ media, demonstrating comparative growth for the cells grown in each media. Panel D shows immunocytochemistry analysis of WA07 hESCs grown in L7^**™**^ hPSC, mTeSR1^**™**^, and E8^**™**^ media, demonstrating comparative expression of OCT4 (red), Nanog (red), SSEA4 (green), TRA1-60 (green), and TRA1-81 (green) in each media. Following differentiation of hPSCs into embryoid bodies (Panel E), differentiated WA07 hESCs readily expressed the markers for early ectoderm (detected TUJ1, green), endoderm (detected Alpha-Feto Protein (AFP), green), and mesoderm (detected Smooth Muscle Actin (SMA), green) in L7^**™**^ hPSC and E8^TM^ media. Cell nuclei are shown by DAPI (blue).

To examine the proliferation of hESCs and hiPSCs cultured in the defined, xeno-free EOD hPSC medium, we compared this medium (hereafter called L7^™^ hPSC EOD medium) to two other feeder-free or xeno-free growth media: PSC mTeSR1^**™**^ and Essential 8^**™**^. To compare the rate of cell expansion, we followed the recommended protocol for each medium including the use of its cell culture system components. The medium comparison study was carried out with WA07 and LiPSC ER2.2. Starting from the same population of cells maintained in L7^**™**^ hPSC EOD medium, hESCs and hiPSCs were passaged into L7^**™**^ hPSC EOD, mTeSR1^**™**^ and Essential 8^**™**^ media and serially subcultured for 5 passages (5–7 days per passage) and underwent cell count evaluation at the end of each passage and further characterization at the end of the study. The data demonstrate that the defined, xeno-free L7^™^ hPSC EOD medium comparably supports the expansion of undifferentiated hESCs and hiPSCs passage by passage, expression of pluripotency markers, and differentiation into ectoderms, endoderm, and mesoderm when compared to mTeSR1^**™**^ and Essential 8^**™**^ (Fig 7 and S2 Fig). While both L7^**™**^ hPSC EOD medium and Essential 8^**™**^ successfully demonstrated EB formation, human PSCs serially subcultured in mTeSR1^**™**^ failed to generate EBs even after incorporating AggreWell^**™**^ (StemCell technologies) in our hands. Lack of EB formation in mTeSR1^**™**^ medium could have been resulted from the complex nature of medium and exposure to relatively high concentration of growth supplements prior to the EB formation step. It may be possible to modify the EB formation protocol to induce EB formation in mTeSR1^**™**^, particularly knowing that the EB formation is a subjective method and modifying the number of days during differentiation could result in different differentiation outcome.

### Generation and expansion of clinical-grade hiPSCs

To substantiate the utilization of the defined xeno-free L7^™^ hPSC EOD medium in the generation of clinical-grade hiPSCs, we derived multiple hiPSC lines from different human tissue sources using the defined, xeno-free L7^™^ hPSC EOD medium. One hiPSC line was derived from dermal fibroblasts, two lines were derived from CD34^+^ cord blood cells, and one line was derived from peripheral blood mononuclear cells (PBMCs). All four hiPSC lines generated from these somatic cell populations displayed morphology comparable to hESCs: colonies with defined edges that contained a high proportion of cells with the prototypically high nucleo-cytoplasmic ratio. Further analysis of cell markers by immunocytochemistry and FACS demonstrated that these hiPSCs express high levels of the pluripotent stem cell markers Oct-3/4, SSEA-4, TRA-1-60 and TRA-1-81, readily differentiate into ectoderm, mesoderm and endoderm, and are genetically stable, as assessed by CGH and SNP analysis (Table 2).

## Discussion

In the present study, we report the development of a defined, xeno-free hPSC medium (i.e. L7^™^ hPSC EOD medium) and its capacity to sustain an EOD schedule of medium replacement for both the generation and long-term cultivation of hPSCs. The robustness of this defined medium formulation allows hPSCs to be reproducibly propagated under conditions where critical supporting supplements and factors are not limiting. This reduces overall variation in the culture and results in the reproducible production of hPSC populations. The option to change the cultivation medium every other day, without compromising the quality of the culture, also lowers the risk of culture contamination. This is particularly valuable in large-scale and clinical grade cultivation of hPSCs, where lost time and the cost of expanding hPSCs *in vitro* can be substantial. Importantly, we have used this medium in combination with L7^**™**^ hPSC passaging solution and L7^**™**^ hPSC Matrix to establish a cGMP-compliant process to generate clinical quantities of pluripotent stem cells [20,24] that can be used in development of clinically relevant materials for cell replacement therapy applications. The L7^**™**^ hPSC medium can comparably support expansion and maintenance of hPSCs *in vitro* when compared to other widely used hPSC cell culture media. Moreover, this xeno-free medium can be further modified into a non-animal origin (NAO) format for manufacturing of hiPSCs or hESCs (data not shown) when animal origin becomes an obstacle for a specific clinical application.

The key factors and supplements identified in formulating this hPSC medium are in agreement with other published reports [5,6,8,11,13,17,25–27]. bFGF plays an important role in promoting and maintaining self-renewal [5,6,8,13], particularly when cultivating hPSCs in defined conditions, where supporting factors are not supplied in serum or by feeder cells. The downstream targets of bFGF signaling, such as mitogen activated proteins kinase (MAPK), phosphoinositide 3-kinase pathways (PI3-K), and Wnt/β catenin, have all been implicated as effectors in the expression of pluripotency associated genes [7,8,10]. Since bFGF is thermodynamically unstable in standard culture conditions [25,28,29], high concentrations of bFGF have traditionally been added to hPSC medium to offset its inherent instability [17–19]. We have tested and determined that TS human recombinant bFGF can sustain the pluripotency of hPSCs at significantly lower concentration and allows using an every-other-day schedule of medium replacement. Replacing bFGF with a TS human recombinant bFGF eliminates the need to introduce excessive amounts of bFGF into the hPSC medium to ensure active bFGF will be present as the culture medium ages over a 24-hour period and as the number of cells present in the culture increases prior to each passage. This effectively decreases the large fluctuations in bFGF concentration normally experienced by hPSC cultures, reducing culture variation. The prolonged stability and effectiveness of the TS bFGF at 37°C allows lower concentrations of bFGF to be used in the cultivation of hPSCs and eliminates the need for any animal-derived components such as porcine heparin [11,30,31].

Use of the defined, xeno-free L7^™^ hPSC EOD medium in combination with a defined matrix (i.e. L7^**™**^ hPSC L7^**™**^ hPSC Matrix) and passaging solution (L7^**™**^ hPSC Passaging solution) standardizes hPSC cultivation conditions and provides a robust process for the generation of iPSCs from various starting materials. The generation of stable hiPSC lines in this defined, xeno-free EOD medium demonstrates its ability to support reprogramming and the genetic stability of newly generated hiPSCs, which allows one to both generate and cultivate hPSCs in one defined medium formulation and helps simplify and streamline the iPSC manufacturing process. The unique combination of basal medium and growth supplements (with optimized and lowered concentrations of essential components such FGF2) allows generation and propagation of hPSCs in a manufacturing process that is unbiased to differentiation into a specific lineage (data not shown). Incorporating a defined, xeno-free, and reliable cell culture system in the manufacturing of human pluripotent stem cells is a key step in the development of standard protocols for generation of functional cell therapy products for cell restorative therapy applications.

## Supporting Information

S1 Fig hPSCs cultured for over 40 passages using the defined, xeno-free hPSC medium differentiate readily to ectoderm, mesoderm and endoderm (Teratoma formation). Human pluripotent stem cells were serially subcultured in L7^**™**^ hPSC cell culture system and exhibited expression of the cells from different lineages. WA09 (H9) hESCs (control cells expanded on feeder system) at passage level 40 exhibited intestinal crypts (IC), smooth muscle bundle (SMB), neural rosette, and cartilage (first row). WA07 (H7) hESCs at passage level 49 exhibited respiratory epithelium (RE), smooth muscle cells (SMC), neural rosette (NR), and cartilage (C) (second row). WA09 (H9) hESCs at passage level 52 exhibited ciliated respiratory epithelium (CRE), primitive mesenchyme (PM), skin adnexal structures (SAS), cartilage, and adipose tissue (AT) (third row). Human iPSCs (NL5) at passage level 53 exhibited IC, bone matrix, and retinal pigment epithelium (RPE), and intestinal goblet cells (IGC), (fourth row). Human iPSCs (LiPSC 18R) at passage level 51 IGC, CRE, SMB, and primitive brain neuropil (PBN) (fifth row).
(TIF)

S2 Fig Comparison of the defined, xeno-free L7^™^ hPSC EOD medium to mTeSR1^™^ E8^™^ medium. Human iPSC line LiPSC ER 2.2 was serially subcultured for five passages. The number of viable cells per passage were compared by initially seeding 2×10^4^ viable cells per cm^2^ into three wells of a six-well plate for each medium, using respective passaging solution and matrix. Panel A shows cell attachment and growth of LiPSC ER 2.2 iPSCs at the end of passage 1 in L7^**™**^ hPSC, mTeSR1^™^ E8^™^ media. Panel B shows cell attachment and growth of LiPSC ER 2.2 iPSCs at the end of passage 5 in L7 hPSC, mTeSR1^™^, and E8^™^ media. Panel C Panel A shows the viability and total viable cells of LiPSC ER 2.2 iPSCs grown in L7^**™**^ hPSC, mTeSR1^™^, and E8^™^ media, demonstrating comparative growth for the cells grown in each media. Panel D shows immunocytochemistry analysis of LiPSC ER 2.2 iPSCs grown in L7^**™**^ hPSC, mTeSR1^™^, and E8^™^ media, demonstrating comparative expression of OCT4 (red), Nanog (red), SSEA4 (green), TRA1-60 (green), and TRA1-81 (green) in each media. Following differentiation of hPSCs into embryoid bodies (Panel E), differentiated LiPSC ER 2.2 iPSCs readily expressed the markers for early ectoderm (detected TUJ1, green), endoderm (detected Alpha-Feto Protein (AFP), green), and mesoderm (detected Smooth Muscle Actin (SMA), green) in L7 ^™^ hPSC and E8^™^ media. Cell nuclei are shown by DAPI (blue).
(TIF)
