# Supplementary figures and images for: A Newly Defined and Xeno-Free Culture Medium Supports Every-Other-Day Medium Replacement in the Generation and Long-Term Cultivation of Human Pluripotent Stem Cells
Source: PLoS One. 2016 Sep 8;11(9):e0161229. doi: 10.1371/journal.pone.0161229 (PMC5016087; doi:10.1371/journal.pone.0161229)

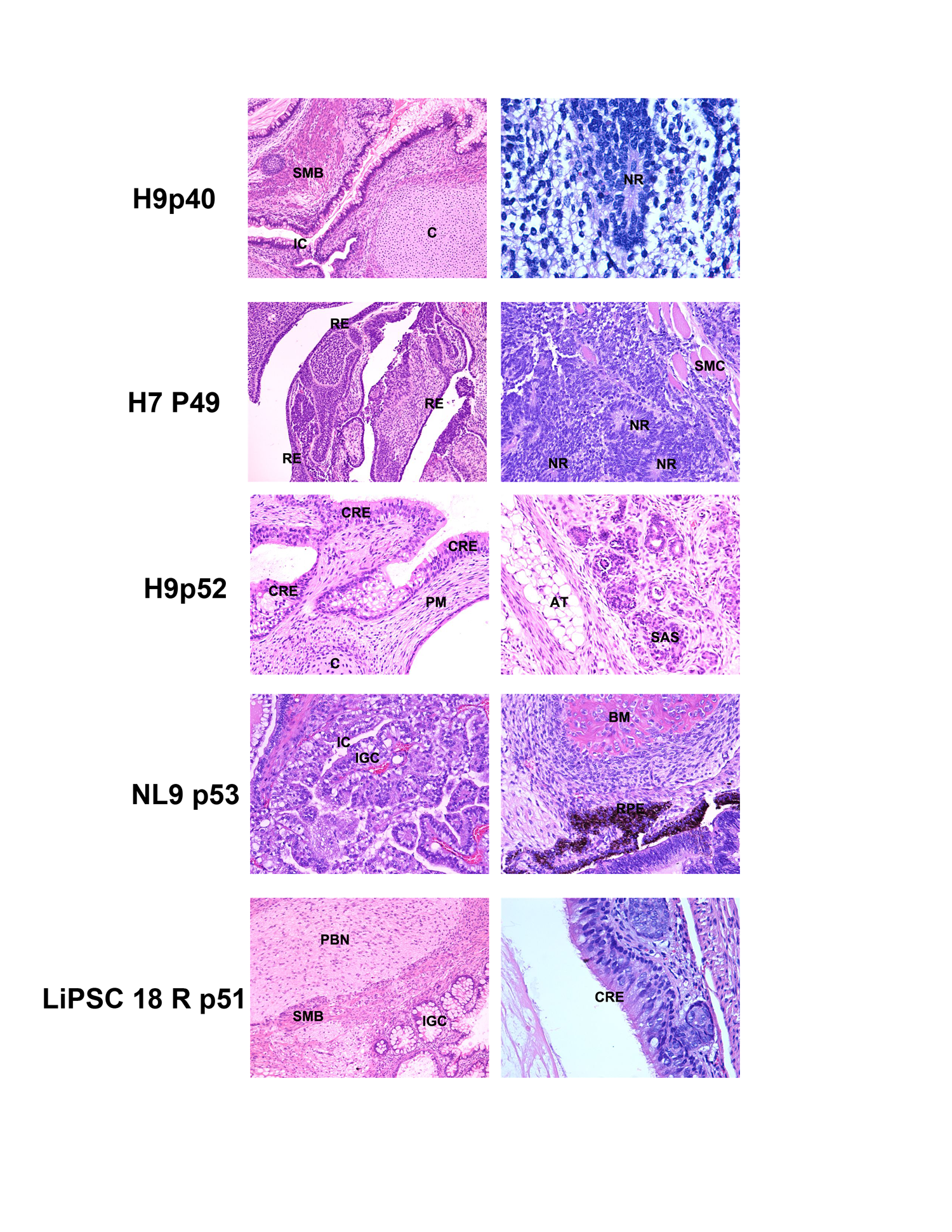

Supplement: S1 Fig — Human pluripotent stem cells were serially subcultured in L7™ hPSC cell culture system and exhibited expression of the cells from different lineages. WA09 (H9) hESCs (control cells expanded on feeder system) at passage level 40 exhibited intestinal crypts (IC), smooth muscle bundle (SMB), neural rosette, and cartilage (first row). WA07 (H7) hESCs at passage level 49 exhibited respiratory epithelium (RE), smooth muscle cells (SMC), neural rosette (NR), and cartilage (C) (second row). WA09 (H9) hESCs at passage level 52 exhibited ciliated respiratory epithelium (CRE), primitive mesenchyme (PM), skin adnexal structures (SAS), cartilage, and adipose tissue (AT) (third row). Human iPSCs (NL5) at passage level 53 exhibited IC, bone matrix, and retinal pigment epithelium (RPE), and intestinal goblet cells (IGC), (fourth row). Human iPSCs (LiPSC 18R) at passage level 51 IGC, CRE, SMB, and primitive brain neuropil (PBN) (fifth row). (TIF) [file pone.0161229.s001.tif]

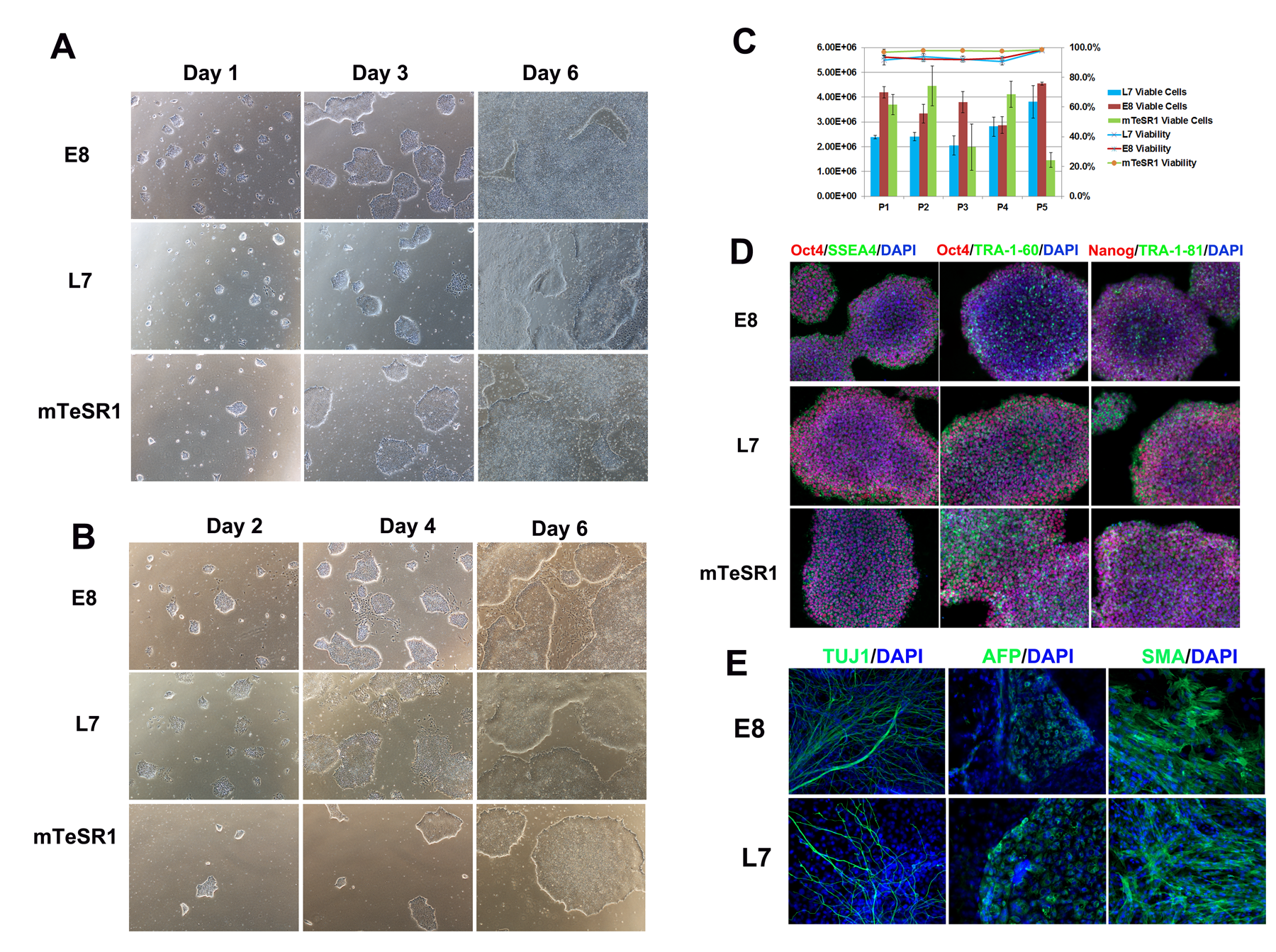

Supplement: S2 Fig — Human iPSC line LiPSC ER 2.2 was serially subcultured for five passages. The number of viable cells per passage were compared by initially seeding 2×10^4^ viable cells per cm2 into three wells of a six-well plate for each medium, using respective passaging solution and matrix. Panel A shows cell attachment and growth of LiPSC ER 2.2 iPSCs at the end of passage 1 in L7™ hPSC, mTeSR1™ E8™ media. Panel B shows cell attachment and growth of LiPSC ER 2.2 iPSCs at the end of passage 5 in L7 hPSC, mTeSR1™, and E8™ media. Panel C Panel A shows the viability and total viable cells of LiPSC ER 2.2 iPSCs grown in L7™ hPSC, mTeSR1™, and E8™ media, demonstrating comparative growth for the cells grown in each media. Panel D shows immunocytochemistry analysis of LiPSC ER 2.2 iPSCs grown in L7™ hPSC, mTeSR1™, and E8™ media, demonstrating comparative expression of OCT4 (red), Nanog (red), SSEA4 (green), TRA1-60 (green), and TRA1-81 (green) in each media. Following differentiation of hPSCs into embryoid bodies (Panel E), differentiated LiPSC ER 2.2 iPSCs readily expressed the markers for early ectoderm (detected TUJ1, green), endoderm (detected Alpha-Feto Protein (AFP), green), and mesoderm (detected Smooth Muscle Actin (SMA), green) in L7 ™ hPSC and E8™ media. Cell nuclei are shown by DAPI (blue). (TIF) [file pone.0161229.s002.tif]
